# Breast feeding and the weekend effect: an observational study

**DOI:** 10.1136/bmjopen-2015-010016

**Published:** 2016-07-08

**Authors:** Emla Fitzsimons, Marcos Vera-Hernández

**Affiliations:** 1 UCL Institute of Education, London, UK; 2 Institute for Fiscal Studies, London, UK; 3 Department of Economics, University College London, London, UK

**Keywords:** breastfeeding, weekend, postnatal

## Abstract

**Objective:**

To compare the incidence of breast feeding by day of week of birth.

**Design:**

Retrospective database study using 16 508 records from the 2005 and 2010 Infant Feeding Surveys.

**Setting:**

England and Wales, UK.

**Participants:**

Mothers of a sample of births from among all registered births in the periods August–September 2005 and August–October 2010.

**Main outcome measure:**

Incidence of breast feeding after birth.

**Results:**

Among babies of mothers who left full-time education aged 16 or under, the incidence of breast feeding was 6.7 percentage points lower (95% CI 1.4 to 12.1 percentage points) for those born on Saturdays than for those born on Mondays–Thursdays. No such differences by day of week of birth were observed among babies of mothers who left school aged 17 or over.

**Conclusions:**

Breastfeeding policy should take into account differences in breast feeding by day of week of birth, which are apparent among low-educated mothers. Further research is needed to ascertain the reason for this finding.

**Strengths and limitations of this study**

- We use data from the 2005 and 2010 Infant Feeding Surveys, the most recent ones available.
- Statistical significance is adjusted using Bonferroni corrections.
- Breastfeeding incidence is self-reported and hence subject to recall bias, although questionnaires were sent to mothers when their children were aged ∼6 weeks.
- Response rates to the Infant Feeding Survey in England and Wales were 61.8% in 2005 and 52.5% in 2010, although weights provided by the survey adjust for non-response.
- Day of week of birth is missing for 1469 children in the 2010 Infant Feeding Survey.

## Introduction

An extensive literature documents the potential benefits of breast feeding for infant health.^1–10^ These benefits might extend to the long term.^11^ Breast feeding is associated with lower blood pressure and lower risk of type 2 diabetes and obesity, as well as with higher cognitive development measures.^7^ ^12–21^ Alongside this, there is a strong socioeconomic pattern in breast feeding. In the UK in 2010, the incidence of breast feeding was 91% among babies whose mothers left full-time education when they were over 18, compared with 75% among those whose mothers left full-time education aged 17 or 18 and 63% among those whose mothers were 16 or under when they left full-time education.^22^

Weekend excess mortality is well documented for emergency admissions, including stroke, trauma, kidney and cardiovascular emergencies.^23–30^ Although such a ‘weekend effect’ might be due to differences in case mix between weekend and weekday admissions, most studies suspect it is due to the decreased availability of experienced healthcare professionals on weekends. Some mothers benefit from the support of hospital staff to initiate and successfully establish breast feeding.^31–44^ We conducted a retrospective study of breast feeding in the years 2005 and 2010, comparing breast feeding incidence rates by day of week of birth. We postulated that breast feeding may vary by day of week of birth, especially for the babies of the least educated mothers, who are less likely to have access to other sources of support and information not provided at hospital.

## Methods

### Data

This paper uses the 2005 and 2010 Infant Feeding Surveys (IFS 2005, 2010).^22^ ^45^ The IFS is a national survey of infant feeding practices carried out every 5 years since 1975 and the main source used to record breast feeding statistics. We attempted to access day of week of birth for 2000, but these data were not available. The IFS contains, among other things, information on the prenatal period (check-ups, classes, intentions on feeding methods, smoking, drinking and nutritional supplement intake), birth experience and the early postnatal period (delivery method, details on breast milk and infant formula milk intake and how the latter is prepared, and support at home), health during the early weeks, introduction of solid foods, intake of additional drinks and supplementary vitamins and basic sociodemographics.

For each country of the UK, unclustered samples of births were drawn from birth registration records containing births that occurred in the periods August–September for the 2005 survey and August–October for the 2010 survey. The surveys were administered via post using a paper questionnaire. Mothers whose children were included in the sample were sent by post an introductory letter, questionnaire and reply-paid envelope, followed by a reminder letter a week later. Up to two more mailings were sent to those mothers who did not reply. The dispatch of the initial questionnaire was staggered on a weekly basis to ensure it reached the mother when the baby was aged ∼4–10 weeks for the 2005 survey and 6 weeks for the 2010 survey. In 2010, there was also the option, for the first time, to fill out the questionnaire online. In each survey, three stages of data collection were conducted, with stage 1 carried out when babies were aged ∼4–10 weeks, stage 2 when they were aged ∼4–6 months and stage 3 when they were aged ∼8–10 months. This paper uses data from stage 1 to measure the incidence of breast feeding.

This analysis uses data from the 2005 and 2010 surveys for England and Wales, which sampled 13 287 births in 2005 and 18 990 in 2010. Of those sampled, 8210 and 9969 completed the stage 1 questionnaire in 2005 and 2010, respectively, yielding response rates of 61.8% and 52.5%, respectively. The total numbers of births for England and Wales were 645 835 in 2005 and 723 165 in 2010.^46^ ^47^

The variable ‘day of week of birth’ was obtained on request from TNS BMRB (for 2005) and IFF Research (for 2010).

### Statistical analysis

The primary outcome of interest is the incidence of breast feeding after birth. The incidence measures the percentage of babies who were breast fed initially, including all babies who were put to the breast at all, even if only once. It also includes babies who were given expressed breast milk. As in the official survey reports, the incidence of breast feeding is measured from the first stage of each survey.^22^ ^45^

We excluded from the analysis 1469 babies whose day of week of birth was not available in the 2010 survey, 170 whose mother's education status was not reported, 24 whose mother's age was not known and 8 whose breastfeeding status was not known. The final sample size is 16 508.

Using weighted logistic regression, we examined the relationship between day of week of birth and our primary outcome (ever breast fed). Proportions were obtained using the estimated parameters averaged across the sample. The breast feeding variable takes the value 0 if the mother reports that the ‘baby has never been given breast milk or been put to breast’ and 1 otherwise. The analysis pools the 2005 and 2010 data sets. Weighted logistic regression controls for the year of survey (2005 vs 2010), type of delivery (normal vs other), maternal age in categories (under 20, 20–24, 25–29, 30–34, 35 or over), country (Wales vs England) and ethnicity (white vs other). Statistical analysis was conducted using Stata software, V.13.1 (StataCorp. Stata Statistical Software: Release 13. College Station, Texas: StataCorp LP, 2013). Proportions, 95% CIs and p values were obtained using the Stata margins command.

Given the stark differences in breast feeding incidence by maternal education, we hypothesised that the effect of day of week of birth on breast feeding might vary by education status. We investigated this hypothesis by entering an interaction term between day of week of birth and education. The interaction term was statistically significant at the 5% level (p=0.027). The subsequent analysis split the data by education status, where low education includes those who left full-time education aged 16 or under and high education includes those who left full-time education aged 17 or over. Although education is not a direct measure of health literacy, it is a proxy for it and is also positively associated with the mother's ability to access different sources of breast feeding support.

To explore the robustness of the findings, additional logistic regression models were estimated with an expanded set of covariates: a binary variable indicating whether the mother was married/cohabiting, prenatal feeding intention (binary variables for exclusive breast feeding and any breast feeding), prenatal care that included infant feeding discussions (binary variables for check-ups and attendance at prenatal classes), a binary variable indicating whether the mother was informed of the health benefits of breast feeding, binary variables as to whether the baby was in special care and whether the baby was put under a lamp for jaundice, and the baby's length of stay in hospital in hours.

## Results

The rate of incidence of breast feeding among mothers with low education is 62.7%, compared with 85.2% among those with high education.

**Figure 1:**
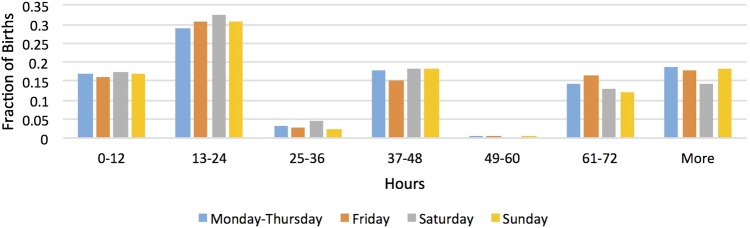
Distribution of length of stay by day of birth for low-educated mothers. Source: 2005 and 2010 Infant Feeding Surveys.

**Figure 2:**
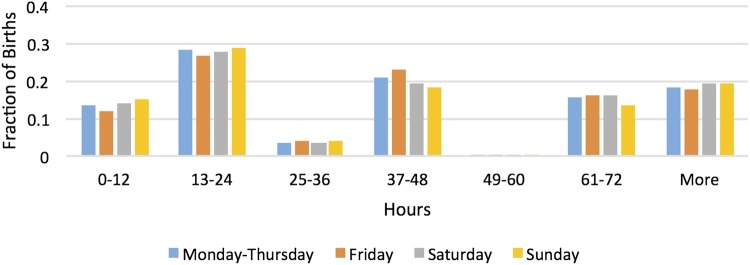
Distribution of length of stay by day of birth for high-educated mothers. Source: 2005 and 2010 Infant Feeding Surveys.

Table 1 shows maternal characteristics by the baby's day of week of birth. It highlights that the distributions of age, age left full-time education, ethnicity, type of delivery and length of hospital stay are similar across days of week of birth. Figures 1 and 2 show the lengths of the baby's hospital stay across days of week of birth for low-educated and high-educated mothers, respectively. The observed pattern likely reflects the facts that babies tend to be born at night and discharged during the day and that hospital discharge policy does not vary by the day of the week. The Kruskal-Wallis rank test does not reject the hypothesis that the distribution of length of stay is the same across days of week of birth (p values are 0.173 and 0.159 for low-educated and high-educated mothers, respectively).

**Table 1:** Maternal characteristics by day of week of birth

	Maternal age				
Day of week of birth	<20	20–24	25–29	30–34	35+
Monday	123 (5.4%)	410 (18.0%)	576 (25.2%)	718 (31.4%)	456 (20.0%)
Tuesday	127 (5.4%)	385 (16.3%)	606 (25.7%)	769 (32.6%)	471 (20.0%)
Wednesday	92 (3.9%)	410 (17.3%)	623 (26.3%)	713 (30.1%)	529 (22.3%)
Thursday	109 (4.5%)	415 (17.0%)	636 (26.0%)	781 (32.0%)	503 (20.6%)
Friday	126 (5.1%)	397 (16.1%)	683 (27.7%)	758 (30.7%)	504 (20.4%)
Saturday	110 (5.0%)	362 (16.4%)	598 (27.1%)	685 (31.0%)	452 (20.5%)
Sunday	128 (5.4%)	392 (16.5%)	636 (26.7%)	730 (30.7%)	495 (20.8%)
	Age mother left full-time education		Maternal ethnicity (white=1)	Normal delivery (yes=1)	Mean length of hospital stay (hours)
	16 or under	17 or over			
Monday	549 (24.0%)	1734 (76.0%)	1937 (84.8%)	1659 (72.7%)	52.2
Tuesday	590 (25.0%)	1768 (75.0%)	2011 (85.3%)	1615 (68.5%)	54.6
Wednesday	510 (21.5%)	1857 (78.5%)	2048 (86.5%)	1631 (68.9%)	53.0
Thursday	556 (22.7%)	1888 (77.3%)	2135 (87.4%)	1613 (66.0%)	52.1
Friday	537 (21.8%)	1931 (78.2%)	2115 (85.7%)	1628 (66.0%)	52.5
Saturday	516 (23.4%)	1691 (76.6%)	1935 (87.7%)	1530 (69.3%)	51.4
Sunday	556 (23.4%)	1825 (76.6%)	2081 (87.4%)	1682 (70.6%)	52.1

Data (other than mean length of hospital stay) are n (%) or frequency (%).

Table 2 shows the incidence of breast feeding by day of week of birth and maternal education status. The incidence of breast feeding is very similar across the days of week of birth for the high educated. However, for the low educated, there is a dip in breast feeding for babies born on Friday and Saturday. Table 3 explores this relationship using logistic regression.

**Table 2:** Incidence of breast feeding by day of week of birth and maternal education status

Day of week of birth	Low educated (n=3814)		High educated (n=12 694)	
Monday	549	(61.8%)	1734	(86.0%)
Tuesday	590	(63.2%)	1768	(86.0%)
Wednesday	510	(64.8%)	1857	(85.6%)
Thursday	556	(64.9%)	1888	(83.2%)
Friday	537	(59.5%)	1931	(85.7%)
Saturday	516	(57.1%)	1691	(85.0%)
Sunday	556	(67.5%)	1825	(84.9%)

Data are n (%). Weighted. ‘Low educated’ includes those who left education aged 16 or under. ‘High educated’ includes those who left education aged 17 or over.

**Table 3:** Logistic regression: relationship between day of week of birth and whether mother ever breast fed

	Unadjusted		Adjusted	
Day of week of birth	Low educated	High educated	Low educated	High educated
Friday				
Difference in percentage breast fed (Friday vs Monday–Thursday)	4.2 ppt.	0.5 ppt.	−3.2 ppt.	0.4 ppt.
95% CI of difference	(−9.7 to 1.3)	(−1.6 to 2.6)	(−8.4 to 2.1)	(−1.6 to 2.4)
p Value	0.134	0.639	0.233	0.688
OR	0.837	1.041	0.866	1.036
95% CI of OR	(0.666 to 1.053)	(0.879 to 1.233)	(0.684 to 1.095)	(0.871 to 1.233)
Saturday				
Difference in percentage breast fed (Saturday vs Monday–Thursday)	−6.6 ppt.*	−0.2 ppt.	−6.7 ppt.**	0.3 ppt.
95% CI of difference	(−12.1 to −1.1)	(−2.4 to 2.1)	(−12.1 to −1.4)	(−1.9 to 2.4)
p Value	0.019	0.877	0.014	0.805
OR	0.759	0.986	0.742	1.023
95% CI of OR	(0.605 to 0.953)	(0.827 to 1.176)	(0.587 to 0.938)	(0.854 to 1.225)
Sunday				
Difference in percentage breast fed (Sunday vs Monday–Thursday)	3.9 ppt.	−0.3 ppt.	3.8 ppt.	0.1 ppt.
95% CI of difference	(−1.2 to 8.9)	(−2.4 to 1.9)	(−1.1 to 8.9)	(−2.0 to 2.1)
p Value	0.136	0.788	0.131	0.940
OR	1.187	0.977	1.197	1.007
95% CI of OR	(0.944 to 1.493)	(0.824 to 1.158)	(0.944 to 1.516)	(0.846 to 1.198)
Observations	3814	12 694	3814	12 694

Data from 2005 and 2010 pooled. All statistical inferences control for year of survey, type of delivery (normal vs other), maternal age in categories (under 20, 20–24, 25–29, 30–34, 35 or over), country (Wales vs England) and ethnicity (white vs other). Effects are relative to Monday–Thursday (reference). ‘Low educated’ includes those who left education aged 16 or under. ‘High educated’ includes those who left education aged 17 or over. The table reports the weighted percentage of breastfed babies born on any of Friday, Saturday or Sunday (separate rows) minus the weighted percentage of breastfed babies born on Monday–Thursday, its 95% CI, p value, OR and the 95% CI of the OR. Significance levels include Bonferroni corrections.

*p<0.1/3=0.0333; **p<0.05/3=0.0167.

ppt., percentage points.

Table 3 shows unadjusted and adjusted differences in the weighted percentages of breastfed babies born on any of Friday, Saturday or Sunday with respect to those born on Monday–Thursday, obtained using a logistic regression with incidence of breast feeding as the dependent variable, stratified by education status. The regression compares separately the births taking place on Friday, Saturday and Sunday with the births occurring on Monday–Thursday inclusive. For the high educated, the differences in weighted proportions are very close to zero and not statistically different from zero in any case. For the low educated, on the other hand, the adjusted (unadjusted) breast feeding incidence is 6.7 percentage points (6.6 percentage points) lower for babies born on Saturdays versus those born on Mondays–Thursdays, with the p=0.014 (0.019), which falls below (slightly above) the Bonferroni-adjusted significance level of 0.0167.

We find that 63.7% of babies of low-educated mothers who were born on Monday–Thursday initiate breast feeding, compared with 57.1% of babies born on Saturday. Put differently, for babies of low-educated mothers, being born on Saturday rather than Monday–Thursday decreases their probability of initiating breast feeding by 10.3%.

Using the same survey data, we estimate that 23.44% of births are to low-educated mothers and that 13.05% of births occur on Saturdays. Multiplying these percentages by the absolute difference of 6.7 percentage points and the total number of births in England and Wales in 2010 (723 165), we calculate that 1482 babies a year are not breast fed in England and Wales because they were born on Saturday rather than on Monday–Thursday.

Results of the logistic regressions estimated using an expanded set of covariates are similar to the main results reported in table 3: the difference in the weighted breast feeding incidence for Saturday versus Monday–Thursday is −4.7 percentage points (95% CI −8.3 to −1.0 percentage points, p=0.012) for low-educated mothers and −0.4 percentage points (95% CI −2.0 to 1.1 percentage points, p=0.576) for high-educated mothers. However, the sample sizes were lower (3487 for low-educated mothers and 11 606 for high-educated mothers) due to missing values in the additional covariates included.

## Discussion

We find that for babies of low-educated mothers, being born on Saturday rather than Monday–Thursday decreases their probability of being breast fed by 10.3%. To put this figure in context, it is equivalent to 1482 fewer babies being breast fed per year; it is also comparable with the effect of the Unicef Baby-Friendly Initiative, a breast feeding-focused intervention that increased the probability of initiating breast feeding by 10%.^33^

Our study has several limitations. We do not have control over statistical power as sample sizes are dictated by the IFS. We use data from the 2005 and 2010 surveys, the most recent ones available. Response rates to the IFS were 61.8% in 2005 and 52.5% in 2010, although weights provided by the survey adjust for non-response. Breastfeeding incidence is self-reported and hence subject to recall bias, although questionnaires were sent to mothers when their children were aged ∼6 weeks.

Data on the day of week of birth were missing for 1469 children in the 2010 IFS. However, the percentage of missing records for women with high education is very similar to that for women with low education (14.5% vs 15.2%). Table 4 reports on the statistical association between records with missing day of week of birth and other variables, stratified by education. Using Pearson's χ^2^ test, no statistically significant associations were found for low-educated mothers. Among mothers with high education, white mothers have a smaller frequency of having missing data. Hence, our results for high-educated mothers should be interpreted with extra caution.

**Table 4:** Percentage of records with missing day of week of birth in the 2010 Infant Feeding Survey

Variables	Low educated (%)	p Value	High educated (%)	p Value
Normal delivery	15.3	0.81	14.2	0.48
Caesarean delivery	14.9		14.8	
White	15.3	0.53	13.6	<0.001
Non-white	14.9		18.8	
Maternal age <20	11.3	0.57	18.4	0.08
Maternal age 20–24	14.9		16.4	
Maternal age 25–29	14.5		14.4	
Maternal age 30–34	16.5		13.5	
Maternal age 35+	16.7		14.3	

p Value refers to Pearson's χ^2^ test.

Another limitation of our study is that our data do not contain time of birth. This blurs the effect of the day of week of birth because children born later on Saturday are more likely to still be in hospital on Monday compared with children born early on Saturday, yet this study treats them the same way. We hypothesise that had we had access to data on time of birth, the day of week of birth effect would be larger.

Other studies report a weekend effect on outcomes such as mortality.^23–30^ Although no conclusive reasons behind these differences are reported, most studies suggest that they may be due to lower staffing and service levels at weekends, as well as differences in the case mix of patients at different times of the week. Facing staff constraints at weekends, hospitals may prioritise labour and delivery, to the detriment of breast feeding support in postnatal wards. Extensive research has shown that early support for infant feeding is critical to the initiation and establishment of successful breast feeding.^31–44^ Other reasons for our findings cannot be ruled out. For instance, visits to hospital from relatives may be higher for children born on Saturdays, which might distract from breast feeding counselling.

Friday is the day with the second lowest breast feeding incidence among low-educated mothers. Children born early on Fridays might benefit from breast feeding support services available during weekdays, which would attenuate the weekend effect for those born late on Fridays. Thus, even though the difference in breast feeding incidence between children born on Fridays and children born on Monday–Thursday did not reach statistical significance, it might still be important to make sure that they receive full breast feeding support.

An effect was not found on Sundays. This might be because, given a median hospital stay of 48 hours, mothers are more likely to be in hospital on weekdays (in particular Monday and Tuesday), thereby benefitting from breast feeding counselling available on weekdays. In the sample, there are 556 Sunday births to low-educated mothers in England and Wales. Of these, only 136 babies stayed <24 hours in hospital; hence, the majority of children (420(=556–136) out of 556) born on Sunday were still in hospital on Monday. Moreover, depending on the exact time of birth, some of the 136 babies who stayed <24 hours were not discharged until Monday. Ultimately, there are few babies born on Sundays who are not also in hospital on Monday, which may be the reason that an effect on Sunday was not found.

An effect was not found for mothers with high education levels. They may be more effective at accessing whatever hospital support is available as well as alternative sources of support such as helplines, community services, information leaflets and lactation consultants. They may also be more likely to use antenatal services better and therefore have more information before the delivery.^48^

These findings have important policy implications. Much of the existing literature documenting a weekend effect is focused on mortality. This paper shows that other dimensions of hospital care are also likely to be affected. Moreover, breast feeding can have long-term benefits for health and cognition, and it can bring future savings to the healthcare system. Current policy to promote breast feeding in the UK should take account of these disparities by day of week of birth, especially for low-educated mothers. Subsequent research should investigate whether these disparities are caused by differences in staffing across the week and/or differences in the number or composition of visits paid by friends and relatives.

An important finding is that the day of week of birth only matters for breast feeding for those from less educated backgrounds. Given long-term beneficial effects of breast feeding, this finding suggests that the day of week of birth may play some role in widening disparities in outcomes across socioeconomic groups.

The research showcases the importance of the IFS in monitoring breast feeding and providing important new evidence for policy. Given that the ninth IFS, due in 2015, did not take place, alternative data sources will be required to monitor progress on the findings we report here.

## Conclusions

Among mothers who left full-time education aged 16 or under, the incidence of breast feeding was 6.7 percentage points lower among babies born on Saturdays than among those born on Mondays–Thursdays. No such discrepancies were observed among mothers who were older when they left full-time education. In the absence of a prospective study, further research is needed to ascertain the exact reasons for this finding.
